# Society Notes

**Published:** 1902-02

**Authors:** 


					﻿Society Notes.
The Central Texas Medical Association held its regular
meeting in Belton January loth and 16th (ult.), with a full at-
tendance. It was a most interesting occasion. We are indebted
to our friend, Dr. J. M. Frazer, chairman of Committee of Ar-
rangements, for a full report of proceedings as published in the
Belton Journal. As it is now a back number, we do not reproduce
it. Dr. F. D. Thompson, of Fort Worth, was elected president
and Dr. W. R. Thompson secretary.
The State Association of Health Officers failed to meet
in Austin January 15 th, as per announcement. They will meet
at Dallas May 6, 7, 8 and 9, during the meeting of the State Med-
ical Association. Due notice will be given through the "Red Back”
with program, and all details.
				

